# Covariate adjustment in cluster randomised trials: a practical guide

**DOI:** 10.1136/bmj-2025-084194

**Published:** 2025-10-24

**Authors:** Karla Hemming, Jack Hall, Andrew Copas, Sam Watson, Monica Taljaard, Richard Hooper

**Affiliations:** 1 Institute of Applied Health Research, University of Birmingham, Birmingham B15 2TT, UK; 2 MRC Clinical Trials Unit at University College London, London, UK; 3 Methodological and Implementation Research Programme, Ottawa Hospital Research Institute, Ottawa, ON, Canada; 4 School of Epidemiology, Public Health and Preventive Medicine, University of Ottawa, Ottawa, ON, Canada; 5 Wolfson Institute of Population Health, Queen Mary University of London, London, UK

## Abstract

Covariate adjustment can offer several potential benefits in the analysis of cluster randomised trials. These benefits include increasing statistical precision (ie, narrowing width of confidence intervals), as well as potentially reducing any bias arising from differential identification and recruitment across arms or missing outcome data. This article outlines a guideline for how to choose covariates to include in a prespecified adjustment plan for such trials. Recommendations include adjusting for covariates that have been included in any restricted randomisation; and adjusting for a prespecified set of covariates thought to be prognostic of the outcome, differential recruitment, or outcome missingness. When the prevalence of missing covariate or outcome data are non-negligible, a missing data technique such as multiple imputation (allowing for clustering), cluster mean imputation, or the missing indicator method, is recommended. In a case study, the proposed prespecified analysis plan includes adjustment for minimisation variables as well as four covariates thought to be prognostic of the outcome and potentially related to unblinded identification of participants after randomisation.

Cluster randomised trials (CRTs) are a vital research design, especially when interventions target groups rather than individuals.^1^ ^2^ ^3^ ^4^ In CRTs, entire clusters—such as schools, hospitals, or communities—are randomised to different interventions, making them particularly useful in public health, education, and health systems research. While CRTs are invaluable, they come with specific methodological challenges. Because individuals within a cluster may respond similarly, statistical analyses must account for this intracluster correlation to generate valid results.^5^ ^6^

Covariate adjustment can have an important role in the analysis of CRTs.^7^ Firstly, in trials that use some form of restricted randomisation (eg, stratification or minimisation), covariate adjustment can increase statistical precision.^8^ ^9^ ^10^ Thus, similarly to individually randomised trials, adjusting for any stratification factors can lead to standard errors that are (appropriately) smaller and confidence intervals that are narrower (compared with no adjustment).^11^ ^12^ ^13^ In addition, adjustment for other prognostic variables, especially baseline measures of the outcome, which were not restricted in the randomisation, can offer further similar advantages.^8^ ^9^ ^14^ Furthermore, similar to individually randomised trials, covariate adjustment (for missingness related covariates) can also have a role in resolving potential bias related to missing data, particularly when differential outcome missingness exists across study arms.^15^ ^16^ ^17^

However, covariate adjustment has a potentially more important role in CRTs than in individually randomised trials. Firstly, in some CRTs, unlike individually randomised trials, individuals might be recruited after clusters have been randomised, which can make CRTs at risk of identification or recruitment bias (also known as lack of allocation concealment and sometimes referred to as a selection bias). These biases manifest as differences in the characteristics of individuals in treatment and control arms—and in extreme cases can render the trial more like an observational study.^18^ ^19^ In these settings, covariate adjustment (for recruitment related covariates) can have an important role in reducing bias (ie, provide a point estimate that is closer to the truth).^20^ ^21^ Moreover, with the randomisation performed at the cluster level instead of at the individual level, the number of randomised units tends to be much smaller (tens rather than hundreds) and substantial imbalances in key cluster characteristics (or even complete confounding) become much more likely. Again, covariate adjustment (in tandem with restricted randomisation) can have a role here, by using the covariate data to explain differences across arms, and ultimately increase statistical precision.

Thus, covariate adjustment can have an even more important role in CRTs than in individually randomised trials. However, covariate adjustment in CRTs has many nuances. For example, covariates might be measured at the level of the cluster, the individual, or a mixture of both; thus, there can be important decisions to be made around how the covariates are included.^22^ ^23^ ^24^ Where covariate adjustment is implemented in an attempt to reduce bias, the covariates included in the adjustment should be considered predictive of that bias (eg, recruitment related covariates, or missingness related covariates).^20^ ^21^ ^25^ Furthermore, many different analytical approaches can be implemented to adjust for covariates, and different analytical approaches can change the target of inference.^3^ ^5^ ^26^

**Summary points**

Covariate adjustment in cluster randomised trials can enhance statistical precision and reduce bias from differential recruitment or missing data
This article provides guidelines for selecting covariates, recommending inclusion of those used in restricted randomisation and those prognostic of outcomes or related to recruitment and missingness
Different model based approaches to covariate adjustment in cluster trials are outlined, as well as missing data techniques, such as multiple or cluster mean imputation
The recommendations are illustrated through a case study involving prespecified covariates

## Objectives

In this article, we aim to provide comprehensive practical guidance for when and how to adjust for covariates in CRTs. We focus on two arm, parallel CRTs with continuous or binary outcomes. Although we include these trials with a baseline period where all clusters are in the control condition, we do not consider stepped wedge or other multiple period designs.

Our recommendations are structured around six key questions that arise in developing a statistical analysis plan for a CRT:

- When should covariate adjustment be considered in a CRT?
- How should covariates be selected?
- What are the different analytical approaches?
- What are the different ways of adjusting for covariates?
- How should missing data on covariates be handled in the analysis?
- What is the relevance of the target of inference?

We include a worked case study to illustrate the recommendations. We present the recommendations in tabulated format and illustrate these recommendations as applied to the case study (table 1). The full worked results for the case study can be found in the supplementary material.

**Table 1:** Recommendations on covariate adjustment in cluster randomised trials with case study application

Recommendations		Case study
**Why**		
Covariate adjustment can offer several potential benefits in the analysis of cluster randomised trials	Increase statistical precision: covariates used in the restricted randomisation have to be adjusted for to realise these gains.	20 clusters were randomised using minimisation on three cluster level variables: region (two levels); pretrial rate of IAP use (binary); and pretrial size of the unit (binary).
	*Potentially reduce bias arising from either differential identification and recruitment across arms; or differential missing outcome data.	Trial is unblinded with post-randomisation identification but no direct participant recruitment. Missing data are anticipated to be minimal.
**What**		
The choice of covariates to include in adjustment should be guided by some broad principles	Covariates included in any restricted randomisation should be adjusted.	Analysis will adjust for three cluster level minimisation factors.
	*Adjustment for a prespecified set of covariates believed to be prognostic for the outcome, differential recruitment, or outcome missingness, should be considered.	Available individual level covariates that are likely to be predictive of the primary outcome and/or any differential identification are the four eligibility criteria (risk factors).
	*Design considerations: in designs with post randomisation unblinded identification or recruitment, consider prespecifying the primary analysis with covariate adjustment.	Primary analysis adjusts for individual level covariates as a precautionary approach to reduce bias under the setting that the design had been subject to differential identification of participants across arms.
	*Adjustment for non-prognostic covariates or a large number of cluster level covariates can lead to a decrease in statistical precision (parsimonious approach).	Only three cluster level covariates will be adjusted for and the four individual level covariates that are all known to be prognostic of the outcome.
**How**		
There are several different ways to implement covariate adjustment	*Covariates can be adjusted directly in a conventional regression model, which allows for clustering (ie, generalised linear mixed models or generalised estimating equations); adjusted indirectly by marginal standardisation (useful for estimation of risk differences or relative risks); or adjusted using two stage, cluster level analysis approaches.	Covariates will be accounted for by mixed model regression adjustment using logistic regression and risk difference, calculated via marginalisation with 95% confidence intervals derived from a t distribution with number of degrees of freedom the number of clusters minus 2 minus the number of cluster level covariates included in the model (for most analysis, this is 20−2−3=15). The generalised linear mixed model approach targets the cluster specific effect, an estimand relevant to, for example, a cluster level decision maker considering the question of whether to implement the intervention in their cluster.
	*Adjusting for the cluster level mean of an individual level covariate is not the same as adjusting for the individual level covariate. Both adjustments may be worth considering and adjusting for cluster level means can be advantageous.	With minimal missing data and only 20 clusters, the approach of adjusting for cluster level means as well as adjusting for the individual level covariate would have reduced the degrees of freedom and therefore was not adopted.
	When the prevalence of missing covariate data are non-negligible, a missing data technique such as multiple imputation (allowing for clustering), cluster mean imputation, or the missing indicator method can be valid approaches.	Prevalence of missing covariate data is very low and so no missing data technique needed.
**When**		
Procedural	Prespecify the covariate adjustment plan in the statistical analysis plan.	Primary analysis is prespecified to include adjustment for four individual level binary covariates and three cluster level covariates.
	*Consider sensitivity analyses in case of unanticipated baseline imbalances.	One additional individual level covariate shows some imbalance (spontaneous delivery). A sensitivity analysis adjusts for this covariate, with results being very similar to the primary analysis.

IAP=intrapartum antibiotics as a prophylactic.

* Different to recommendations for individually randomised trials.

## Case study

GBS2 is a parallel group (unblinded) cluster randomised trial of the transmission of group B *Streptococcus* (GBS) from mother to child.^27^ Twenty UK maternity units (20 clusters) were randomised to a strategy of rapid test (intervention) or usual care arm (control). Under the control arm, women were offered intrapartum antibiotics as a prophylactic treatment for GBS. In the intervention arm, only women with a positive test for GBS colonisation were offered intrapartum antibiotics as a prophylactic. The primary objective was to reduce the proportion of women receiving intrapartum antibiotics. Pregnant women were eligible for inclusion if they had one or more of the following risk factors: a previous baby with GBS disease; GBS bacteriuria during the current pregnancy; preterm labour (less than 37 weeks’ gestation); and maternal pyrexia (≥38°C). Data were from routinely collected sources and there was no direct participant recruitment. However, eligible participants were identified after randomisation, which has the possibility of inducing biases. The 20 clusters were randomised by minimisation on three cluster level variables: the region at two levels; the pretrial rate of intrapartum antibiotic use as a prophylactic (binary: categorised as above or below the median); and the pretrial size of the unit (binary: categorised as above or below the median).

## When covariate adjustment should be considered in cluster randomised trials

In CRTs, covariate adjustment can both increase statistical precision (ie, standard errors become smaller and confidence intervals become narrower) and, in some settings, reduce bias (ie, point estimate is closer to the truth). We consider the inclusion of covariates that have been included in a restricted randomisation (to increase statistical precision); additional covariates that are known to be prognostic of the outcome (also to potentially increase statistical precision); and covariate adjustment in settings of differential recruitment or outcome missingness (to potentially reduce bias).

### Any covariates used in a restricted randomisation should be adjusted for in the analysis

Using a form of restricted randomisation (eg, stratification) can increase precision under a model based analysis (eg, a linear mixed model, see below). Therefore, factors used within the restriction need to be adjusted for in the analysis as covariates in order to realise that gain (ie, achieve correct precision for the required treatment effect).^28^ In practice, this means that standard errors from the adjusted analysis are smaller (and likewise confidence intervals are narrower) than those without adjustment.^29^ ^30^ This approach is known to also hold within the context of CRTs.^8^ ^9^ ^10^ ^14^ The implications of failing to adjust for randomisation covariates can be quite substantial: for a trial with a small number of clusters, the gain from covariate adjustment for randomisation covariates can be equivalent to around a 15 point increase in statistical power.^8^ Thus, any covariates included in a restricted randomisation procedure should normally be adjusted for in the primary analysis to increase precision.

### Additional precision can be gained by covariate adjustment for variables not included in the randomisation

In CRTs, covariate adjustment for a small number of additional prognostic covariates (either cluster level or individual level) can also improve statistical precision.^8^ ^9^ For example, chance imbalances between cluster level characteristics can be quite common in CRTs, particularly so for such trials with a smaller number of clusters. Having a prespecified covariate adjustment plan to mitigate this imbalance can be of benefit. In addition, adjustment for individual level covariates (which by their nature cannot be included in a restricted randomisation) can also increase statistical precision.^8^ ^9^ If the covariates selected happen to be non-prognostic, adjustment does not induce bias—but it might reduce statistical precision (see below).^9^ ^31^ ^32^

### Covariate adjustment can reduce bias if recruitment or outcome missingness differ across study arms

If participants are identified or recruited after randomisation, the knowledge of what arm the participant is being recruited to can lead to differential recruitment of participants across study arms.^33^ In these situations, adjusting for recruitment related covariates (ie, those that can predict differential recruitment) in the right functional form can remove this bias, and may reduce such bias otherwise.^20^ ^21^ ^34^ ^35^ In practice, this principle means that point estimates from approaches that adjust for (recruitment related) covariates are closer to the truth. Covariate adjustment can reduce bias, however, under some important conditions—including but not limited to the assumption of a homogenous treatment effect (between those participants recruited and not recruited). Whether these assumptions are tenable is unlikely to be testable in most trials.^34^ Nonetheless, under less restrictive assumptions, covariate adjustment can remove bias and so it is likely to be important.^20^ ^21^ When the covariates that are responsible for the differential recruitment are unknown (or they are incorrectly identified), some efficiency might be lost.^35^

Covariate adjustment can also be important in reducing bias when there is differential outcome missingness, for example, when reasons for missing outcomes differ across study arms. Again, if the measured covariates can explain this differential missingness then covariate adjustment can lead to a reduction in bias.^16^ ^25^ ^36^ ^37^ Thus, where the study design includes unblinded identification or recruitment of participants after randomisation, or missing outcome data that might be differ across arms, adjusting the primary analysis for covariates is likely to be the preferred approach.^7^

## How covariates should be selected

When covariate adjustment is considered necessary or desirable, the next question is how to select covariates to use in the adjustment. Generally, we recommend approaches that prespecify covariates based on perceived prognostic strength (of outcome, differential recruitment, or differential outcome missingness); that different considerations might be needed depending on the study design and execution; and a parsimonious approach that limits in some sense the number of included covariates.

### Selection based on perceived prognostic strength (outcome, differential recruitment, or differential outcome missingness)

Researchers may be tempted to select covariates for adjustment by testing for so-called baseline imbalance (ie, testing for a significant difference between covariates across study arms). However, this approach might lead to inflated type I error^38^ ^39^ and is generally not recommended.^40^ Instead, recommendations mostly suggest a pragmatic approach of choosing a limited set of covariates on the basis of their perceived prognostic strength.^41^ ^42^ ^43^ In CRTs, this selection includes covariates that are predictive of the outcome, of any differential identification or recruitment (recruitment related covariates), and of any missing outcome data (missingness related covariates). In practice, what covariates are prognostic in a particular setting will not always be known with complete certainty, but examples of covariates that might be important are baseline measures of the outcome as well as age, sex, socioeconomic status, severity of condition in question, and comorbidities. One way of eliciting covariates for adjustment is for investigators at the design stage to consider what covariates they would be concerned about if they showed an imbalance and to specify adjustment for these covariates in advance. In situations with an apparent baseline imbalance, and the characteristic in question was not (for whatever reason) included in the prespecified analysis plan as a covariate, we might still consider an unplanned sensitivity analysis that includes it.

### Selection biased on study design and execution

The identification of the threat of bias related to identification and recruitment should not be made by post hoc inspection or statistical testing of the baseline table, but rather be informed by the design features that render this bias a likely concern.^33^ So, for example, in a CRT where the participants are recruited after clusters have been randomised and where those individuals who manage the recruitment are unblinded to the treatment allocation, covariate adjustment for the primary analysis is likely to be very important. Likewise, even in CRTs without any direct participant recruitment, there can still be a threat of identification bias (see case study, above). Chosen covariates should be those thought to be prognostic of differential identification or recruitment across arms. Subject area expertise will likely be important in establishing these covariates.

### Importance of retaining a parsimonious approach

Adjustment for covariates that are not prognostic might decrease statistical precision.^9^ ^32^ When adjusting for covariates that are not prognostic (ie, have little to no association with the outcome), efficiency gains are minimal and, in some cases, such adjustments might even lead to a slight decrease in efficiency owing to the introduction of additional variability without corresponding explanatory power.^31^ Similarly, adjustment for a large number of (relative to the number of clusters) cluster level covariates can also be detrimental (because the degree of freedom reduces by one for every cluster level covariate included^44^). Likewise, misspecification of the association between the outcome and the covariate can also decrease statistical precision.^32^ Thus, while adjustment for covariates not included in the randomisation might increase statistical precision, some care is needed, because adjustment for non-prognostic covariates, misspecifying the functional form of the association between covariates and outcome, or adjustment for many cluster level covariates, can run the risk of decreasing statistical precision (ie, making the confidence intervals wider).

## Analytical approaches of cluster randomised trials

CRTs have two major analytical approaches: individual level analysis and cluster level analysis.^5^ Two commonly used individual level approaches using multivariable regression are generalised linear mixed models and generalised estimating equations.^6^ In CRTs, any approaches should allow for the clustered nature of the data and appropriate small sample corrections in CRTs with fewer than about 40 clusters.^45^ The choice of approach often depends on statistical preference and ease of implementation, but should also consider the estimand of interest (where the estimand is a precise description of what treatment effect is being measured to answer a specific scientific question, see below).

### Direct covariate adjustment using generalised linear mixed models

In this approach, the outcome is modelled at the individual level, and both individual level and cluster level covariates can be included as independent variables in the model.^6^ This is one of the most commonly used approaches.^46^ Generalised linear mixed models (and generalised estimating equations, see below) can be used to either directly adjust for covariates or (less commonly) to indirectly adjust for covariates, for example, using an approach such as marginal standardisation (also known as g-computation) or propensity scores.^47^

### Direct covariate adjustment using generalised estimating equations 

This approach involves modelling the conditional mean of the individual level outcome given the intervention and can accommodate both individual level and cluster level covariates.^6^ Generalised estimating equations are less commonly used than generalised linear mixed models in practice (perhaps because they can have poor statistical properties in small samples), but are likely the preferred approach when estimating a risk ratio via a technique known as modified Poisson regression^48^ or when using marginal standardisation as a way of estimating risk differences (see below).^47^

### Marginal standardisation

Marginal standardisation (also known as g-computation) is a three step procedure for covariate adjustment.^49^ ^50^ In the first step, the covariates are included in a multivariable regression (eg, a logistic model fitted via generalised estimating equations). In the second step, the predicted outcomes from the fitted model (first step) are obtained for every observation in the study sample in each study arm. Finally, these predicted outcomes are averaged (marginalised) across all observations in each arm, and then contrasted (eg, on the log relative risk scale). Standard errors are typically obtained using delta methods.^51^ ^52^ Because logistic regression can have better convergence properties over some other regression methods, this approach can be more stable when trying to estimate relative risks or risk differences, particularly with rare outcomes.^6^ Marginal standardisation is more commonly used in conjunction with generalised estimating equations (when implementing with generalised linear mixed models, there are added complexities of how the random effects should be incorporated when marginalising over the covariates).

### Two stage analysis

In its simplest form under this approach, the outcome is modelled at the cluster level, and cluster level covariates are included as predictors.^3^ ^53^ Cluster level analyses are less commonly used, but can be more precise than individual level methods and potentially robust to model misspecification.^31^ When using a cluster level analysis, the adjustment for cluster level covariates is straightforward, but require an indirect (or two stage approach) to adjust for individual level covariates.^3^ ^54^ The cluster level approach can also be combined with weights (to weight clusters by their size), which can increase statistical precision, but which can also change the estimand of inference (below).^55^

## Different ways of adjusting for covariates

After choosing the general analytical approach, several additional considerations remain, pertaining to different ways of adjusting for covariates. These ways can include adjusting for baseline values of the outcome, whether to adjust for individual or cluster level versions of covariates, and how to accommodate complex relationships such as interactions.

### Different ways of adjusting for baseline values of the outcome

For covariates that represent baseline measures of the outcome, adjustment will almost always increase statistical precision, and this will be the case whether analysing the follow-up scores or change from baseline.^23^ ^24^ Thus, baseline measures of an outcome should be a strong contender for covariate adjustment. However, baseline measures of the outcome can be accommodated in the analysis in different ways, which depend on whether the same participants are repeatedly assessed (cohort design) or different participants assessed at each time point (cross sectional design). There are, however, essentially two ways of performing this adjustment: an analysis of covariance-type approach or in a repeated measures-type model.

For example, in a cohort design, the individual level outcome measure at baseline can be included in the analysis model as a covariate—in an analysis of covariance type approach.^24^ ^56^ In a cross sectional design (with different individuals at baseline and follow-up), we might consider adjusting for the cluster level mean at baseline as a covariate in an individual level analysis of covariance. However, if additional individual level covariates are adjusted for, a two stage approach could be considered. Alternatively (again in a cohort design, but also of relevance in a cross sectional design), the individual outcomes at baseline and follow-up can be included in a generalised linear mixed model using a repeated measures type of model (with appropriate allowance for correlations within individuals). This model can include time (follow-up *v* baseline) and treatment arm by time interaction as fixed effects, with the difference between the randomised arms at baseline constrained to be zero through omission of the treatment arm’s main effect.^24^

### Adjustment for cluster means versus individual level covariates

In CRTs, covariates can be available at the level of the cluster or level of the individual. Examples of cluster level covariates include the type or size of hospital, whereas examples of individual level covariates include individual receipt of free school meals. When a covariate is measured at the level of the individual, it is possible to adjust for the individual level covariate, the cluster level summary, or both. In CRTs where individuals have outcomes assessed at baseline and follow-up, it has been demonstrated that adjusting for the cluster mean of the baseline outcome, in addition to the individual baseline outcome, can increase statistical power.^23^ ^24^ Moreover, adjustment for cluster means is one way (but not the only way) of avoiding having to discard outcome data from individuals who have a missing value for the covariate.^24^ ^56^ Thus, covariate adjustment might take the form of an individual level adjustment, a cluster mean adjustment, or both.

### Modelling the functional form of covariates

The literature on specification of the functional form of the covariate relationship in CRTs is not extensive, but the current literature supports the need to accurately model the association between the outcome and covariate in order to fully realise precision gains.^32^ Where complexities such as interactions or non-linear associations are expected, then propensity score approaches can be more statistically efficient than direct covariate adjustment.^32^ However, despite concerns around the validity of additional assumptions when using covariate adjustment, evidence to date suggests that models are asymptotically robust to model misspecification.^50^ ^57^ Therefore, while accurately modelling the correct form of the association will help realise statistical precision gains, it is not necessary to avoid bias (at least not asymptotically).^57^ Adjusting for treatment-covariate interaction terms to account for treatment effect heterogeneity can further improve precision,^31^ ^57^ and possibly reduce bias.^25^ Marginalisation can then be used to estimate the marginal (ie, overall) effect of treatment if desired.

## How missing data on covariates should be handled in analysis

CRTs that researchers anticipate to have at least some missing data will need a strategy to accommodate this.^58^ The principles by which missing covariate or outcome data should be handled in CRTs largely follow those in individually randomised trials, but with a few differences. In CRTs, missing data might include individual level covariates, individual level outcomes, cluster level covariates, or even entire clusters. Any missing outcome data might be differentially missing across arms. A common approach to handling missing covariate data—namely, an available case analysis (ie, a covariate adjusted analysis that includes only those observations with all covariates and outcome data available)—might not necessarily be the most robust or efficient approach (see below).

### General principles around handling missing covariate data in CRTs

When the percentage of missing covariate data is very low (much less than 5%), an available case analysis is likely to be a reasonable approach. Similarly, in CRTs with a large number of clusters (>50), generalised estimating equations with covariate adjustment is robust under “missing at completely at random” assumptions; whereas generalised linear mixed models can be valid under missing at random assumptions (provided models are not misspecified).^16^ ^59^ In other settings, more robust approaches should be considered—for example, strategies such as multiple imputation^60^ or joint modelling of the outcome and missing data mechanism.^58^ Simple but potentially robust approaches can adjust for the cluster level mean (ie, single mean imputation) or include the missing indicator approach.^56^ ^61^ ^62^ If multiple imputation is used, imputation models should be congenial to the analysis model, meaning that they should include all relevant variables of interest in the analysis, such as cluster indicators, outcomes, treatment indicators, and interactions. They may also include auxiliary covariates (ie, additional covariates not already included in the analytical model and that help explain missingness).^63^ ^64^ Both covariate adjustment in a mixed model type approach and multiple imputation can remove bias induced by differential outcome missingness, so long as an interaction is included between the covariates and intervention.^25^

## Relevance of the estimand of inference

There is an increasing appreciation for the need to carefully prespecify the research question, particularly in terms of the target of inference.^30^ When estimating non-collapsible measures of effect (eg, odds ratios or hazard ratios), covariate adjustment changes the effect being estimated from a marginal to covariate-conditional treatment effect.^43^ A related consideration in CRTs is whether the estimand of interest is the cluster specific effect or a cluster marginal effect. Cluster specific effects are conditional on belonging to a particular cluster, while cluster marginal effects consider the impact of the intervention on a population level summary aggregated over all clusters and individuals.^65^ ^66^ The two effects can differ when estimating non-collapsible measures of effect. The cluster marginal effect is useful when the goal is to estimate the overall effect of an intervention across the entire target population (eg, when the intervention is intended to be applied broadly), and when the findings aim to inform general policy or practice decisions.

## Discussion

Covariate adjustment can have two important roles in the analysis of cluster randomised trials: it can improve statistical precision and potentially reduce bias. Any covariates that have been included in any restricted randomisation should usually be adjusted for in the primary analysis. Additional covariates considered for adjustment should be based on their perceived prognostic strength and anticipated association with differential recruitment or outcome missingness, while being mindful that adjustment can decrease precision where covariates are not prognostic. The selection of these covariates should usually be by prespecification. In CRTs with unblinded recruitment after randomisation of clusters, or in settings where a non-negligible amount of missing outcome data is anticipated, covariate adjustment is likely to be of greater importance and should be considered for the primary analysis.

Covariate adjustment can be implemented in several different ways, including conventional regression models; indirect adjustment through marginal standardisation; or two stage approaches. Of course, researchers should account for the clustered nature of the trial in any analysis approach. Individual level covariates can also be aggregated into cluster level means for adjustment, with or without adjustment for the individual level version of the outcome. When outcome or covariate data are missing, it can be possible for inference to be robust and efficient without any special missing data approach—for example, by adjustment for cluster level means or single mean imputation.

Our focus is to provide practical recommendations to enhance the appropriateness of analyses of CRTs with respect to covariate adjustment. With this in mind, we have tried to find a balance between practicality and complexity of statistical methods. Recommendations are in line with international guidance encouraging the routine adjustment for covariates used in the randomisation and for other covariates to be prespecified.^67^

### Context—how covariate adjustment is used contemporary CRTs

In CRTs, adjustment for covariates that have been included in a restricted randomisation (eg, stratification) will improve statistical precision compared to the unadjusted effect.^8^ ^9^ ^10^ ^14^ Covariate adjustment thus helps in the quest for efficient use of resources. However, most CRTs do not adjust for covariates used in the randomisation.^68^ ^69^

Furthermore, researchers should consider the requirement to report unbiased estimates of treatment effects. In CRTs, bias can arise for many reasons, but bias from post randomisation identification or recruitment of participants^19^ ^33^ or from differential missing outcomes^16^ ^37^ can be important. Conducting CRTs with recruitment of participants after randomisation is not uncommon (only 30% of CRTs conducted in schools have participants recruited before randomisation^70^). In these settings, covariate adjustment can have an important role in protecting against bias—yet adjusting for covariates is relatively uncommon in CRTs.^71^

In addition, because many CRTs are conducted using routinely collected data and might therefore be prone to missing covariates (or indeed outcomes), strategies are required to accommodate any missing covariates.^72^ Yet, while many CRTs have missing data, most have an available case analysis.^16^ ^37^ ^73^

### Future role of machine learning

Some recent developments in CRTs have not been considered here. These novel strategies help mitigate the loss in precision that can arise when adjusting for non-prognostic covariates.^74^ ^75^ Machine learning approaches have also been proposed to model the non-linear effects of covariates^50^ ^74^; and doubly robust estimators have been proposed to accommodate missing covariates.^17^ ^62^ ^75^ ^76^

### Gaps in methodological literature on guidance

Much methodological work is still needed to verify if the recommendations made here hold in all settings and to identify situations where there may be exceptions. For example, the need to adjust for covariates used in the randomisation to improve statistical precision has been demonstrated in the context of individually randomised trials^29^; for continuous and binary outcomes in CRTs when estimating the odds ratio using generalised linear mixed models^8^ ^9^; and when using a two stage approach.^75^ Whether these recommendations hold under more general models, for example, with survival or count outcomes, remain to be established.^74^

Other questions include whether to consider the original cluster level version of covariates used in a restricted randomisation, or consider a more relevant version at the individual level. For example, in a school based CRT, the proportion of students having free school meals (cluster level covariate) might have been used in the restricted randomisation. At the analysis stage, however, information might be available on that covariate at the individual level. Some researchers have recommended that if a covariate has been used in the randomisation as a cluster level mean but is available in the individual level form for the analysis, then it should be entered into the analysis model in its individual level form.^9^ Given the breadth of different approaches, there are also many specific and nuanced questions around the analysis approach (eg, whether, when using a cluster level analysis, a multiple imputation model should be used on the individual level data followed by aggregation).

### How to estimate standard errors

We did not consider standard error and degree of freedom specification in the presence of covariate adjustment.^77^ In practice, many of the analysis approaches for cluster data have to be combined with small sample standard error corrections or used with degree of freedom corrections in settings with fewer than about 40 clusters. Furthermore, which standard error correction or degree of freedom to use depends on context.^77^ The approach to small sample correction can differ with and without covariate adjustment.^9^ For example, on adjustment for cluster level covariates, one candidate degree of freedom is the number of clusters minus the number of cluster level covariates; whereas the number of degrees of freedom do not reduce on individual level adjustment.^44^ The use of robust variance, which is relatively common when using generalised estimating equations, is another potential approach for standard error correction and has also been recently recommended within the context of mixed models,^73^ and is recommended by some researchers for use with covariate adjustment more generally.^43^

### Alternative approaches not considered

Some other approaches to covariate adjustment have not been considered here, including propensity scores, which can also be used in CRTs.^78^ Propensity scores are a two step approach.^20^ ^32^ In the first step, a model is fitted so that the covariates are used to predict the propensity for being in the treatment or control arm (perhaps counterintuitively, without a random effect for cluster). In the second step, the propensity scores can be used either via direct adjustment for the score to give a covariate conditional effect, or used to derive weights to give a marginal covariate effect.^32^ In the context of individually randomised trials, propensity score approaches have been shown to be sometimes less efficient that direct covariate adjustment and the same seems to apply in CRTs.^21^ ^79^ We also did not consider all the different potential approaches to marginal standardisation in cluster trials. For example, the average could be defined as the contrasts across all individuals, or defined as an average of cluster averages; marginalisation could be conducted within both the context of generalised estimating equations and generalised linear mixed models; or within the context of generalised linear mixed models, the marginal estimate could be produced either by fixing the random cluster effect to be zero, at the best linear unbiased estimates of the random effects for each cluster, or by marginalising over the random effects.

### Importance of estimands

Among many reasons to urge triallists to use covariate adjustment in analysis, there are nuances. For example, direct covariate adjustment typically estimates a covariate conditional effect rather than a marginal effect.^43^ Moreover, the typical approaches to covariate adjustment in CRTs (ie, generalised linear mixed models and generalised estimating equations) have different targets of inference.^66^ Indeed, in CRTs, estimation using generalised linear mixed models (which estimates the cluster specific effect) is much more common than using marginal models (an almost 10-fold difference).^16^ ^73^ We have not entered the debate on which target estimand should be the preferential target for any given setting. Whether the marginal effect or covariate conditional effect is more relevant in individually randomised trials is under debate.^80^ ^81^ ^82^ In CRTs, these issues are more complex. For example, where intervention effects vary by clusters due to their size, it can be particularly important to choose an analysis approach that targets the estimand of interest—but guidance or consensus is limited on whether cluster level or individual level averages are most suited in any given research study in CRTs.^26^ Thus, despite a recent drive to encourage trialists to choose an analysis approach that matches their target of inference, there is likely a need for improved clarity of recommendations, and additional education regarding different possible targets of inference and when they might be of broad interest.^83^

### Conclusion

Covariate adjustment has an important role in CRTs, both improving statistical precision and potentially reducing biases. Any covariate adjusted analysis should usually be prespecified in any statistical analysis plan for a CRT. This prespecified plan requires careful consideration, particularly with regard to the analytical approach, the functional form of the covariates, the choice of the covariates, and how missing covariates will be accommodated. Covariate adjustment will not always be sufficient to enable unbiased intervention effects in the setting of post-randomisation identification or recruitment biases. Thus, we underscore the need to use strategies to prevent differential identification or recruitment or missing outcome data.

## Web extra

Extra material supplied by authors

Web appendix: Supplementary Material case study

## Notes

Dissemination to participants and related patient and public communities: There is no plan to disseminate the information as there were no research participants included in this study.

Provenance and peer review: Not commissioned; externally peer reviewed.
