# Supplementary material for: Covariate adjustment in cluster randomised trials: a practical guide
Source: BMJ. 2025 Oct 24;391:e084194. doi: 10.1136/bmj-2025-084194 (PMC12550654; doi:10.1136/bmj-2025-084194)
Supplement: Supplementary file 1 — Web appendix: Supplementary Material case study [file hemk084194.ww.pdf]

Supplementary Material Case study: Rapid intrapartum test for maternal group B streptococcal colonisation and its effect on antibiotic use in labouring women with risk factors for early-onset neonatal infection (GBS2)

Background

GBS2 is a parallel-group (unblinded) cluster randomised trial [Daniels 2022]. UK maternity units (20 clusters) were randomised to a strategy of rapid test (intervention) or usual care arm (control). Under the control arm women were offered Intrapartum Antibiotics as a Prophylactic (IAP) treatment for GBS. In the intervention arm, only women with a positive test for GBS colonisation were offered IAP. The primary objective was to reduce the proportion of women receiving intrapartum antibiotics. Identification of eligible participants was done after randomisation which has the possibility of inducing biases.

Here we present an illustration of a statistical analysis plan focusing on covariate adjustment using the principles outlined in this paper (noting this was not the planned analysis for this trial). We start with a brief summary of the relevant aspects of the trial, outline and justify the plan for covariate adjustment, illustrate and interpret findings.

Eligibility criteria

Pregnant women were eligible for inclusion if they had one or more of the following risk factors: (i) a previous baby with GBS disease; (ii) GBS bacteriuria during the current pregnancy; (iii) pre-term labour (less than 37 weeks' gestation); and (iv) maternal pyrexia (≥ 38 °C). Data were from routinely collected sources and there was no direct participant recruitment.

Randomisation

Randomisation of the 20 clusters was implemented using minimisation on three cluster-level variables ((i) region – two levels; (ii) pre-trial rate of IAP use (binary – categorised as above or below the median); and (iii) pre-trial size of the unit (binary – categorised as above or below the median)).

Covariates available

The following individual-level covariates were additionally available for consideration: age (years); onset of labour (spontaneous or induced); type of delivery (vaginal, instrumental, caesarean); multiparity (yes, no); and each of the maternal risk factors listed above in the inclusion criteria.

Covariate adjustment analysis plan

The analysis will adjust for the three cluster-level minimisation factors: region (categorical, two levels); pre-trial rate of the primary outcome and number of caesarean sections (both measured in the pre-trial period and adjusted for in their continuous form).

Whilst it is not expected that the eligibility criteria would induce any differential recruitment across the arms and missing outcomes are likely to be minimal, under a pre-cautionary approach and because even in the absence of any bias, covariate adjustment should improve statistical precision, the analysis will adjust for a small number of additional covariates. To choose covariates for adjustment we considered which of the available individual-level covariates are likely predictive of the primary outcome and/ or any differential recruitment. The four eligibility criteria ('risk factors') are anticipated to be the most prognostic and so are included in the plan as covariates for adjustment. In summary, this means a plan to adjust for four individual-level binary covariates and three cluster-level covariates (two of which are continuous). This is labelled the primary analysis approach in the tables.

In the absence of any strong evidence that the effect of any of the continuous covariates is non-linear they are adjusted in their continuous form assuming a linear effect. The percentage of missing covariate data was anticipated to be negligible. Covariates will be accounted for using mixed model regression adjustment using logistic regression and then a difference calculated through marginalisation. With only a small number of clusters we report 95% confidence intervals derived from a t-distribution with number of degrees of freedom the number of clusters minus two minus the number of cluster-level covariates included in the model (for most analysis this is thus 20- 2-3= 15). Note we use a GLMM approach and so target the cluster-specific effect, an estimand relevant to for example a cluster-level decision maker considering the question of whether to implement the intervention in their cluster (for

this reason, when estimating the risk difference by marginalisation we set the cluster random effect to be zero – so as to target an effect for the typical cluster).

Sensitivity analysis

In the event of strong imbalance on covariates not pre-selected for adjustment, face validity would be undermined without adjustment. Thus, where there is a clear indication of imbalance on any covariates, in a sensitivity analysis those fully covariates would be added into the adjusted model. Any none pre-specified sensitivity analysis should be considered exploratory. Those which align with primary analysis can be considered supportive; whereas those that do not align might be important in overall interpretation.

Implementation

The results presented are from Stata 18. Code is provided for both Stata and R implementation. Delta standard errors are presented for marginal standardisation. The analysis excludes the very small amount of missing data.

Results

The baseline characteristics of the clusters and individuals, stratified by arm are presented in Table 1. There are some evident differences between covariates across the study arms, for example: the proportion with GBS bacterium detected in the current pregnancy is 329/721 (46%) vs. 332/906 (37%). There is one covariate that appears imbalanced which was not planned for adjustment in the primary analysis: the percentage with a spontaneous delivery is 343/697 (49%) vs. 527/891 (59%).

The planned primary analysis, (adjustment for the minimisation variables and the four risk factors): gives an OR: 0.82 (0.47 to 1.46) and RD: -0.02 (-0.10 to 0.05), both non-statistically significant and with a point estimate favouring rapid test. It is noted that the estimated effects from the unadjusted analysis and the analysis adjusted only for the minimisation variables, have a point estimate favouring usual care and have wider CIs for the risk difference (note the CIs for ORs should not be compared directly, as they are all targeting slightly different estimands). Thus, adjustment for covariates appears to both increase statistical precision and might even be correcting for bias (given the value of the point estimate changes direction).

After adjusting for the one additional covariate that exhibits some imbalance (as per the sensitivity analysis), the values hardly change. This finding lends support to the overall primary analysis result unlikely to be confounded (or biased) due to the imbalance in this one covariate.

References

[Daniels 2022] Daniels JP, Dixon E, Gill A, Bishop J, Wilks M, Millar M, Gray J, Roberts TE, Plumb J, Deeks JJ, Hemming K, Khan KS, Thangaratinam S; GBS2 Collaborative Group. Rapid intrapartum test for maternal group B streptococcal colonisation and its effect on antibiotic use in labouring women with risk factors for early-onset neonatal infection (GBS2): cluster randomised trial with nested test accuracy study. BMC Med. 2022 Jan 14;20(1):9. doi: 10.1186/s12916-021-02202-2. PMID: 35027057; PMCID: PMC8759240.

Stata and R code

Software program	Code
Stata	xtset Cluster<br>xtlogit y i.Treatment x1 x2 i.x3 x4, re<br>lincom 1.Treatment, df(15) or<br><br>margins Treatment, post<br>nlcom _b[1.Treatment] - _b[0.Treatment], df(15)
R	require(marginme)<br>require(glmmTMB)<br>fit <- glmmTMB(y ~ Treatment + x1 + x2 + x3 + x4 + (1|Cluster), data = df, family = binomial(link="logit"),REML = TRUE)<br>m <- margin(fit,<br>x = "Treatment",

	type = "diff",<br>average = c("x1","x2","x3","x4"),<br>re = "zero",<br>se="GLS")<br>summary(m)<br>confint(m, df = 15)

Variables: y = the outcome indicator, binary; Treatment = the treatment indicator, categorical; Cluster_= cluster identifier, numeric; x1, x2, x4 = continuous covariates (cluster level or individual level); x3 = binary covariate (cluster level or individual level)

Inference uses a between-within small sample correction by specifying the degrees of freedom. In the example this is the number of clusters (20) minus the number of cluster level covariates (4 – 3 covariates plus intercept) minus 1.

Table 1: Baseline characteristics for the GBS2 trial

	Rapid test	**Usual care**
**Cluster-level covariates**	**N = 10**	**N = 10**
**Cluster region used in minimisation**		
Region A	5 (50.0)	5 (50.0)
Region B	5 (50.0)	5 (50.0)
**Baseline IAP**		
Below median	6 (60.0)	4 (40.0)
Above median	4 (40.0)	6 (60.0)
Median [IQR]	25.3 [22.8 to 32.6]	27.5 [9.9 to 31.7]
**Size of cluster**		
Below median	5 (50.0)	5 (50.0)
Above median	5 (50.0)	5 (50.0)
Median [IQR]	4539 [3567 to 5583]	4000 [2930 to 5050]
**Individual-level covariates**	**N = 721**	**N = 906**
**Woman's age (years)**		
Mean (SD), N	29.3 (5.83), 721	30.1 (5.78), 906
Missing	0	0
**Type of labour**		
Spontaneous	343 (49%)	527 (59%)
Induced	354 (51%)	364 (41%)
Missing	24	15
**Type of delivery**		
Spontaneous Vaginal	439 (61%)	542 (60%)
Instrumental	102 (14%)	131 (14%)
Emergency Caesarean	173 (24%)	233 (26%)
Missing	7	0
**Previous pregnancy**		
No	255 (35%)	321 (35%)
Yes	465 (65%)	585 (65%)
Missing	1	0
**Risk factors for neonatal GBS infection**		
Previous baby with neonatal GBS disease	51 (7%)	53 (6%)
GBS bacterium detected in current pregnancy	329 (46%)	332 (37%)
Maternal temperature ≥38 whilst in labour	66 (9%)	156 (17%)
Preterm labour	325 (45%)	431 (48%)

Table 2: Estimated treatment effects of Rapid test vs. Usual care on the use of Intrapartum Antibiotics

Outcome and Model	Rapid test	Usual care	Estimate (95% CI); SE[1] Rapid test vs Usual care
**Use of Intrapartum Antibiotics**	297/716 (41%)	328/906 (36%)	
GLMM – unadjusted			OR: 1.11 (0.62 to 1.99); 0.276
			RD: 0.02 (-0.10 to 0.15); 0.061
GLMM – adjusted for minimisation variables (categorical form)			OR: 1.28 (0.80 to 2.07); 0.224
			RD: 0.06 (-0.05 to 0.16); 0.050
GLMM – adjusted for minimisation variables (continuous form)			OR: 1.15 (0.68 to 1.93); 0.244
			RD: 0.03 (-0.08 to 0.15); 0.054
GLMM* – adjusted for minimisation variables (continuous form) and risk factors			OR: 0.82 (0.47 to 1.46); 0.267
			RD: -0.02 (-0.10 to 0.05); 0.034
GLMM^ – adjusted for minimisation variables (continuous form), risk factors, and onset of labour			OR: 0.81 (0.47 to 1.42); 0.260
			RD: -0.03 (-0.10 to 0.04); 0.033

*Planned primary approach ^Sensitivity analysis An OR less than 1 means rapid test better.

[1]Standard error of the log odds ratio and risk difference
